# State-Dependent Gain Modulation of Spinal Motor Output

**DOI:** 10.3389/fbioe.2020.523866

**Published:** 2020-10-02

**Authors:** Robert Guggenberger, Valerio Raco, Alireza Gharabaghi

**Affiliations:** Institute for Neuromodulation and Neurotechnology, Department of Neurosurgery and Neurotechnology, University of Tüebingen, Tüebingen, Germany

**Keywords:** sensorimotor integration, state-dependent stimulation, closed-loop stimulation, neuroprosthesis, brain-machine interface, neurorehabilitation

## Abstract

Afferent somatosensory information plays a crucial role in modulating efferent motor output. A better understanding of this sensorimotor interplay may inform the design of neurorehabilitation interfaces. Current neurotechnological approaches that address motor restoration after trauma or stroke combine motor imagery (MI) and contingent somatosensory feedback, e.g., via peripheral stimulation, to induce corticospinal reorganization. These interventions may, however, change the motor output already at the spinal level dependent on alterations of the afferent input. Neuromuscular electrical stimulation (NMES) was combined with measurements of wrist deflection using a kinematic glove during either MI or rest. We investigated 360 NMES bursts to the right forearm of 12 healthy subjects at two frequencies (30 and 100 Hz) in random order. For each frequency, stimulation was assessed at nine intensities. Measuring the induced wrist deflection across different intensities allowed us to estimate the input-output curve (IOC) of the spinal motor output. MI decreased the slope of the IOC independent of the stimulation frequency. NMES with 100 Hz vs. 30 Hz decreased the threshold of the IOC. Human-machine interfaces for neurorehabilitation that combine MI and NMES need to consider bidirectional communication and may utilize the gain modulation of spinal circuitries by applying low-intensity, high-frequency stimulation.

## Introduction

In patients with severe and persistent motor deficits after trauma or stroke, motor imagery (MI) and feedback technology are being investigated as a potential therapeutic intervention to activate the motor system, enhance corticospinal excitability and restore function (Stevens and Stoykov, 2003). The concept of these interventions is to achieve plasticity and functional recovery on the basis of contingent activation of natural efferent and afferent pathways (Biasiucci et al., 2018). The underlying neurophysiological mechanisms of these interventions are, however, still under investigation. One potential mechanism is the increase of corticospinal gain modulation (Khademi et al., 2018, 2019; Naros et al., 2019). The focus of previous studies has been on the induced changes at the cortical level (Gharabaghi, 2016), although there is some research on spinal changes following intervention at the lower limb (Takahashi et al., 2019).

MI engages motor cortical areas similar to those engaged in actual motor practice, via, for example, sensorimotor event-related desynchronization (ERD; Pfurtscheller and Neuper, 1997; Lotze et al., 1999; Neuper et al., 2005; Miller et al., 2007, 2010; Kaiser et al., 2011). MI has been shown to enhance ERD (Reynolds et al., 2015) and increase corticospinal excitability (CSE) to a greater extent in combination with neuromuscular electrical stimulation (NMES) than without NMES, thereby, reaching levels similar to those occurring during voluntary muscular contraction (Kaneko et al., 2014). These CSE increases were related to intracortical processes mediated via GABA_A_ergic (Abbruzzese et al., 1999; Stinear and Byblow, 2004; Takemi et al., 2013) and GABA_B_ergic disinhibition (Chong and Stinear, 2017) and may thus serve as the pre-synaptic input for an excitatory drive via proprioceptive input (Kraus et al., 2016a).

In addition, MI may fulfill the requirements of associative stimulation (Hebb, 1949; Harel and Carmel, 2016) by modulating an extended cortical motor network and its susceptibility to additional stimulation (Vukelić et al., 2014; Bauer et al., 2015; Vukelić and Gharabaghi, 2015a,b). Accordingly, previous studies have shown that pairing specific brain states with peripheral (Mrachacz-Kersting et al., 2012, 2016), cortical (Kraus et al., 2016b) or combined stimulation (Gharabaghi et al., 2014a; Royter and Gharabaghi, 2016; Kraus et al., 2018) increased corticospinal excitability and achieved motor gains (Naros and Gharabaghi, 2015; Naros et al., 2016; Belardinelli et al., 2017). Cortical motor mapping with refined transcranial magnetic stimulation protocols (Kraus and Gharabaghi, 2015, 2016; Mathew et al., 2016) provided further insight into the differential modulation of sensorimotor areas by these neurofeedback interventions (Kraus et al., 2016a; Guggenberger et al., 2018).

Self-regulation and neurofeedback of cortical beta-band oscillations are therefore being investigated as novel methods for facilitating associative plasticity and motor restoration in stroke patients with persistent motor deficits (Naros and Gharabaghi, 2015; Belardinelli et al., 2017). Specifically, motor imagery with contingent proprioceptive feedback via passive movement by a robotic orthosis is applied to activate the motor system (Bauer et al., 2015) and enhance the oscillatory beta modulation range in the absence of overt movement (Vukelić et al., 2014; Vukelić and Gharabaghi, 2015a) with a proportional increase of corticospinal connectivity (Gharabaghi et al., 2014b; Kraus et al., 2016a) and motor gains (Naros and Gharabaghi, 2015; Naros et al., 2016).

Knowledge on the effects of MI on spinal excitability is less detailed and still a matter of some debate. Some studies have demonstrated a facilitatory effect of motor imagery on spinal excitability (Rossini et al., 1999; Taniguchi et al., 2008; Ichikawa et al., 2009; Hara et al., 2010; Fujisawa et al., 2011; Takemi et al., 2015), whereas others have not (Abbruzzese et al., 1996; Kasai et al., 1997; Hashimoto and Rothwell, 1999; Facchini et al., 2002; Patuzzo et al., 2003; Sohn et al., 2003; Stinear et al., 2006). For the lower limb, there is evidence that MI combined with peripheral stimulation may modulate reciprocal inhibition, while the H-reflex was not affected (Takahashi et al., 2019). This discrepancy may be partly related to methodological differences such as the number of stimuli or the probing technique applied, i.e., direct and indirect stimulation of the spinal motor neuron via the F-waves and the H-reflex, respectively (Takemi et al., 2015).

In the present study, we took a different approach of measuring the impact of afferent input by applying NMES and probing the stimulation effects with kinematic recordings of the induced wrist deflection to capture the overall spinal motor output. We hypothesized that the state-dependent (MI vs. REST) increase of spinal excitability is sensitive to the intensity and frequency of neuromuscular stimulation. We explored two different frequencies of stimulation (30 vs. 100 Hz). The rationale underlying the use of 30 vs. 100 Hz stimulation was that the higher frequency would induce more afferent input due to indirect and mechanically driven excitation of the Golgi tendon organ (Aguiar and Baker, 2018). Furthermore, central contributions are believed to recruit primarily slow-twitch fibers (Dean et al., 2007), which are already recruited directly by 30 Hz stimulation (Wyndaele, 2016; Vromans and Faghri, 2018). Therefore, we expected to see stronger deflection effects for 100 vs. 30 Hz stimulation.

## Materials and Methods

### Participants

Twelve healthy volunteer subjects (6 females, age: 23–33 years, all right-handed), participated in the study. The subjects reported no previous history of surgery involving the upper limb. All subjects had normal or corrected-to-normal vision. After being instructed about the experimental procedure, the subjects provided written informed consent. The study was approved by the local ethics committee and carried out in accordance with the principles of the Declaration of Helsinki.

### Experimental Set Up

Electromyographic (EMG) recordings were obtained from BrainAmp system (Brain Products GmbH). Bipolar EMG recordings were performed on the extensor carpi radialis (ECR) of the right hand, using Ag/AgCl adhesive electrodes (Ambu GmbH). All signals were sampled at 5,000 Hz and digitized with 16 bits. A kinematic glove (VHAND 3.0, DGTech Engineering Solutions), equipped with an inertial measurement unit (IMU) on the dorsal side of the hand was used for kinematic recordings. Kinematics were acquired with a sampling frequency of 30 Hz and digitized with 12 bits, providing a resolution of 0.01°. Right wrist extension was measured as the roll angle provided by the IMU. NMES was applied using a Rehamove2+ stimulator (Hasomed GmbH) on the right forearm of the subjects. Stimulation was applied using a pair of round self-adhesive electrodes (diameter 50 mm, Zen-Qui) positioned over the muscle belly and tendon of the ECR.

### Experimental Protocol

The experimental procedure was preceded by a preparation phase where individual hotspots and stimulation intensities were determined for each subject.

#### Preparation Phase

During this phase, the subjects sat on a chair placed in front of a screen with their right arm resting on a surface and their hand hanging completely relaxed. A pillow was positioned under the forearm to ensure that the subject was comfortable for the whole duration of the experiment. The room was kept quiet and dimly illuminated to reduce the effect of any auditory or visual stimuli other from those delivered as instructions during the experiment. Prior to the beginning of the experimental session, the subjects were verbally instructed about the task. The instruction entailed the experimental procedure, the task, and the stimulation effects. The participants were specifically instructed to keep their right arm relaxed throughout the experiment. The difference between kinesthetic and visual MI was explained to them and they were requested to perform the former (Darvishi et al., 2017). Subjects were instructed to perform MI of wrist extension only during the imagery phase and to continue even when stimulation was applied. A short familiarization session was performed before starting the actual experiment. This enabled the subjects to experience the trial structure and imagery tasks. While the subjects were receiving instructions about the task, a second experimenter prepared the skin prior to electrode placement using abrasive gel and alcohol. The stimulation electrodes were placed on the arm to induce a functional extension of the wrist, minimizing the finger contribution. EMG electrodes were positioned above the ECR muscle close to the muscle tendon and belly without touching the stimulation electrodes.

After the kinematic glove had been set up, a preliminary range of stimulation intensities was screened to detect (a) the current intensity required to obtain a visible deflection of the wrist from the resting position and (b) the current intensity required to produce a maximum extension, given the mechanical constraints of the wrist. Subjects were asked to provide feedback about noxious sensations during this screening. One subject reported discomfort at intensities below the plateau intensity and had to be excluded from the study. The preliminary screening was followed by a complete screening in steps of 0.5 mA starting at the intensity that was found to produce a functional movement of the wrist. Each intensity was delivered in bursts of 3 s stimulation with 1 s breaks and repeated five times for each frequency. Biphasic pulses (pulse width 800 μ*s*) were applied under constant-current control. The pulse-width was selected to maximize the contribution of the spinal reflexes to the evoked muscle contraction. The recorded displacement of the wrist during the stimulation train was used to draw kinematic IO curves of the two frequencies. We calculated the 5th and 95th percentile of the curve to estimate the upper and lower plateau. Furthermore, we fitted a three parameters sigmoidal function to the IO curves:

(1) $$f\,\left(i\right)=A÷\left(1+e^{\left(\theta +i\right)\times \beta }\right)$$

where *i* represents the stimulation intensity, *A* represents the range of the curve and resembles the upper plateau, θ the threshold and β the slope of the curve. For each frequency, nine amplitudes were selected to allow us to sample the shape of the IO curve during the experiment. By default, the stimulation intensities were set at the threshold θ as determined by this initial assessment, and ±4 steps of 0.5 mA. In cases where the resulting stimulation interval would not cover the whole IO curve, the nine stimulation intensities were set at three intervals of ±0.5 mA centered at threshold and at intensities achieving the two plateaus.

#### Experimental Phase

**FIGURE 1:**
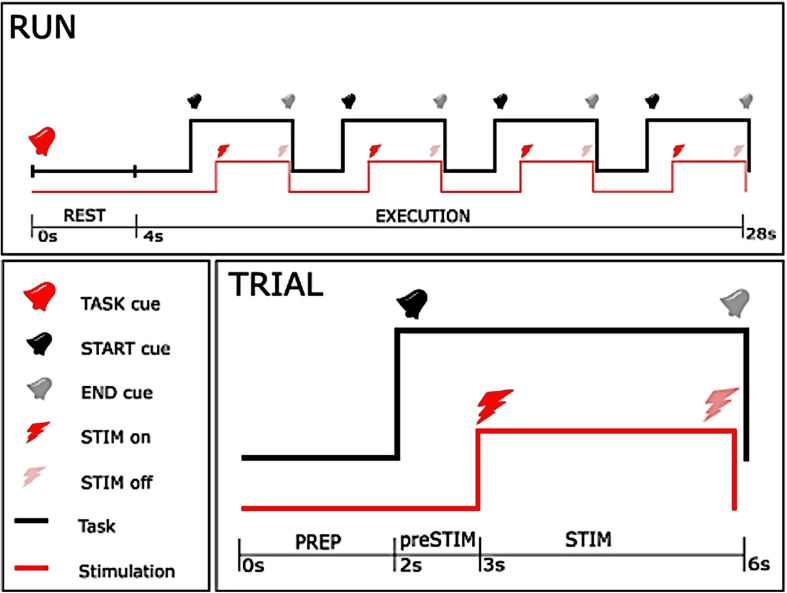
Time-course of the experiment. The upper figure shows the time-course of each single run, with the lower right figure showing the course of a single trial. As can be seen from the upper plot, a 24 s execution period with one specific instruction (either rest or motor imagery) started after a 4 s rest period, and consisted of 4 trials. In each trial, stimulation started 1 s after the start cue, and stopped 3 s later concurrently with the end cue with 2 s between trials.

The experiment consisted of 5 sessions, each composed of 18 runs (Figure 1). A pause of 5 min was taken between consecutive sessions to avoid muscular fatigue due to the electrical stimulation. Each run was characterized by one of the two task instructions, either rest (REST) or motor imagery (MI), and began with a 4 s rest period, at the beginning of which an auditory cue instructed the subject about the task to be performed (TASK cue). The rest period was followed by four trials, composed of a 2-s preparation epoch (PREP) and a 4-s execution epoch, separated by auditory cues (START and END cue). After the 1st second of the execution phase, a 3-s stimulation train was delivered to the subject.

The stimulation was composed of a high frequency (30 or 100 Hz) square biphasic pulse at one of the nine intensities established during the preparation phase for each stimulation frequency. Task instruction (REST or MI), frequency (30 or 100 Hz) and intensity of stimulation (one out of nine predefined intensities) were randomized across the entire session. Randomization allowed us to reduce a possible bias due to order effects, e.g., caused by muscular fatigue or improvement of motor imagery in the course of the intervention. The whole procedure consisted of 360 stimulation bursts, where each condition of frequency and task was repeated 10 times for every intensity in a 2 by 2 factorial design. Every participant took part in all conditions, i.e., stimulation at 30 and 100 Hz, and motor imagery vs. no imagery. The duration of the experimental phase was less than 50 min, and total duration of stimulation was 18 min.

### Data Analysis

#### Volitional Contractions

To ensure that there was no voluntary movement before the stimulation, a *t*-test between the conditions REST and MI was conducted on the initial value of the joint angle. Additionally, we analyzed EMG data acquired pre-stimulation and during stimulation periods to ensure that there was no voluntary movement. During the pre-stimulation period, the EMG signal was zero-phase filtered using a band-pass third order Butterworth digital filter with 10 Hz and 500 Hz as lower and upper cut-off frequencies, respectively. A notch third order Butterworth filter was used to remove the power supply frequency, i.e., 50 Hz, and the second and third harmonic frequencies (i.e., 100 and 150 Hz). Subsequently, the mean absolute value (MAV) of the pre-processed signal was calculated. During the stimulation period, only the artifact-free periods between stimulation pulses were analyzed. The volitional EMG activity was extracted by estimating the electrically induced potential (M-wave) and its removal through an adaptive filtering procedure on the basis of the repeatability of the M-wave and on the Gaussian amplitude distribution of the volitional EMG signal as described in Sennels et al. (1997) and Ambrosini et al. (2014). The MAV of the estimated volitional EMG signals was then calculated.

A *t*-test was conducted for each subject and each stimulation amplitude to assess whether there was any significant difference between the rest and motor imagery condition. The statistical analysis was performed for each stimulation intensity to assess whether the amplitude of stimulation influenced the potential presence of voluntary contraction. Subjects who presented voluntary contraction were excluded from further analyses.

#### Kinematic Analysis

The range of the angular wrist positions was recorded by the kinematic glove during the task and served as a measure of wrist displacement. To evaluate the effect of MI on the NMES induced wrist deflection, a sigmoidal function identical to the one used during the screening (see section “Preparation Phase”) was fitted to the data. We estimated the three parameters slope β, threshold θ, and saturation level *A* for the IO curve of each subject, for each task and frequency. A three-way analysis of variance (ANOVA) was run for each of these parameters with the categorical factors FREQUENCY and TASK, the random factor SUBJECT, as well as for the interaction between TASK and FREQUENCY. We repeated the ANOVA with a permutation test with 1,000 repetitions, to ensure that our results were not affected by non-normality. Additionally, we performed *post-hoc t*-tests following the analysis of variance.

## Results

### Volitional Contraction

One participant reported discomfort at intensities below the plateau intensity and was excluded from the study. Another participant showed a significant increase in MAV prior to MI and was excluded from analysis. Therefore, this data analysis was performed in 10 out of 12 participants. For these participants, the measured wrist angular displacements were not caused by voluntary contraction, and could reliably and specifically be attributed to the NMES-induced contraction with and without MI.

### Kinematic Results

**FIGURE 2:**
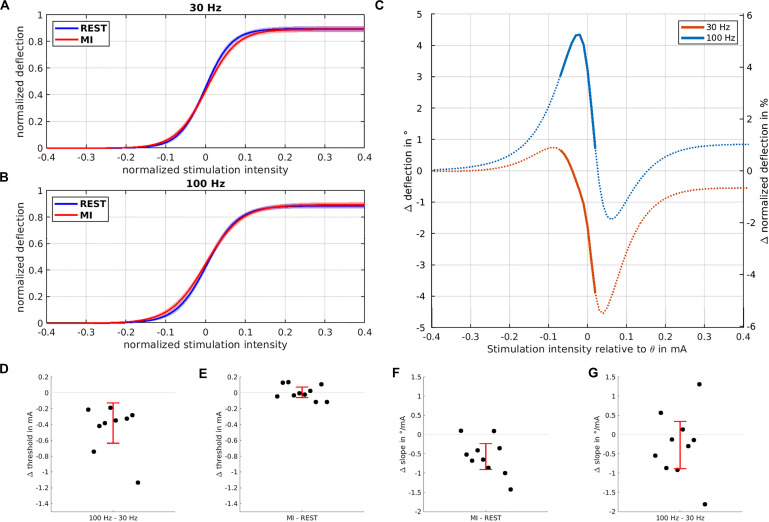
Influence of stimulation intensity and motor imagery on curve parameters. The normalized input-output curves based on the sigmoidal model fit and averaged across all subjects are shown for 30 Hz **(A)** and 100 Hz **(B)** highlighting the kinematic deflection when NMES was applied during REST (blue trace) vs. MI (red trace). **(C)** Modulation of the input-output curves induced by MI for 30 Hz (blue trace) and 100 Hz (orange trace) stimulation; most pronounced wrist deflection occurs at subthreshold intensities for 100 Hz stimulation. Significant differences between the two frequencies are marked by thick lines. The threshold is significantly reduced **(D)** when stimulation is performed at 100 vs. 30 Hz, but not for MI vs. REST **(E)**. The slope of the IOC **(F)** is significantly reduced when stimulation is performed during MI vs. REST, but not for 100 vs. 30 Hz **(G)**. In all four lower plots **(D–G)**, each dot indicates a single subject, with the red error bars showing the 95% confidence interval of the mean difference.

The IOC exhibits a clear sigmoidal shape for both stimulation frequencies, during both rest and MI (Figures 2A,B). Having fitted the sigmoidal model to the IOC, the ANOVA of the IOC parameters shows that *A*, the maximal deflection (*M* = 76.9°, *SD* = 20.45°) was not influenced by TASK or FREQUENCY (all *p* > 0.24, permutation test all *p* > 0.23). Yet, the threshold θ (*M* = 9.66 mA, *SD* = 1.75 mA) was affected by FREQUENCY [*F*(1, 27) = 30.56, *p* < 0.001, permutation test *p* < 0.001], with a higher threshold for 30 Hz (*M* = 9.85 mA) compared to 100 Hz (*M* = 9.47 mA) (Figure 2D), while no such effect was found for MI versus REST (Figure 2E). Moreover, the slope [β (M = 2.96^°/*mA*^, *SD* = 1.3°^/mA^)] was influenced by TASK [*F*(1, 27) = 9.03, *p* = 0.0057, permutation test *p* = 0.005], disclosing a lower slope for MI (*M* = 2.58°^/mA^) compared to REST (3.25°^/mA^) (Figure 2F), while no such effect was found for 100 vs 30 Hz. We found no evidence for interactions on any IOC parameter (all *p* > 0.66, permutation test all *p* > 0.63), suggesting independence of TASK and FREQUENCY effects. Qualitative inspection of the effect of MI on the IOC for both stimulation frequencies suggested that the deflection was enhanced at subthreshold intensities (Figure 2C). *Post-hoc t*-test analysis disclosed a significant increase of wrist deflection during MI at subthreshold intensities, but only when stimulation was delivered at 100 Hz [*t*(9) = 2.76, *p* = 0.022].

## Discussion

This work showed that motor imagery increased spinal motor output at low NMES intensities, while the responsiveness of spinal motorneurons was differently modulated by the frequency of the afferent input (100 vs. 30 Hz).

Unlike in previous studies, which applied nerve stimulation to probe MI-related spinal excitability by the corresponding reflexes (Abbruzzese et al., 1996; Kasai et al., 1997; Hashimoto and Rothwell, 1999; Rossini et al., 1999; Facchini et al., 2002; Patuzzo et al., 2003; Sohn et al., 2003; Stinear et al., 2006; Taniguchi et al., 2008; Ichikawa et al., 2009; Hara et al., 2010; Fujisawa et al., 2011; Takemi et al., 2015), we used NMES to acquire an input-output (IO) curve of the induced muscle contraction and probed two distinct frequencies.

Application of this technique enabled us to disentangle the influence of MI on different mechanisms that generate spinal motor output: Muscle contraction can be induced by direct excitation of the muscle, and by indirect, central mechanisms. The latter may comprise (i) antidromic activation of motor axons, (ii) activation of sensory axons providing excitatory synaptic input to spinal neurons that recruit motor units (Collins et al., 2001, 2002; Dean et al., 2007), and (iii) mechanic excitation of proprioceptors, e.g., the Golgi tendon organs (Aguiar and Baker, 2018).

Previously, the central mechanism was maximized by applying high-frequency NMES (100 HZ) while avoiding antidromic block by stimulating at a relatively low intensity (Collins et al., 2002; Dean et al., 2007). The present work extended this line of research by demonstrating that this central mechanism may be amplified by MI and supraspinal contributions: MI reduced the IO slope, i.e., increased the responsiveness of spinal motorneurons to low intensity NMES. High-frequency (100 vs. 30 Hz) stimulation contributed to this mechanism by decreasing the stimulation intensity at which the inflection point of the IO curve occurred, so that the combination of MI and low-intensity 100 Hz increased the overall NMES-induced spinal motor output.

We speculate that the reduced threshold during 100 Hz stimulation is caused by increased afferent input, likely via the Golgi tendon organs (Aguiar and Baker, 2018). Furthermore, it is plausible that the reduced slope that occurs during stimulation with both frequencies is caused by supraspinal priming.

This might explain why a considerable number of previous studies did not find a facilitatory effect of motor imagery on spinal excitability. They either probed the peripheral contribution (Facchini et al., 2002; Patuzzo et al., 2003; Sohn et al., 2003; Stinear et al., 2006) or applied different stimulation parameters (Abbruzzese et al., 1996; Kasai et al., 1997; Hashimoto and Rothwell, 1999; Patuzzo et al., 2003). The present work could not determine whether MI activated higher threshold motoneurons and/or motoneuron plateau potentials (Bennett et al., 1998; Dean et al., 2007); a question that needs to be answered in future studies. Moreover, the additional effect sizes induced by MI were significant but rather small, which is probably related to the already strong spinal activation by NMES. Future research needs therefore to clarify whether repetitive pairing of MI and NMES, as specified here, will result in relevant increases of excitability and even plastic modulation on the spinal level as well. Future work may also research whether MI may be replaced by motor attempts to prime spinal motoneurons. Such attempts may, however, be difficult to perform by healthy subjects when they are simultaneously asked to avoid overt movements.

In any case peripheral input such as NMES may be paired with different paradigms of oscillatory cortical stimulation to achieve lasting effects on corticospinal excitability via associative plasticity (McNickle and Carson, 2015; Guerra et al., 2016; Nakazono et al., 2016; Raco et al., 2016, 2017; Naros and Gharabaghi, 2017). The present work is, however, limited when it comes to disentangling direct (monosynaptic) and indirect (oligosynaptic) contributions of cortical activity on spinal excitability with the methods applied here. Future work will need to include additional measures such as the latencies of evoked potentials in response to stimulation to answer this question. Moreover, the ability to perform motor imagery is known to be variable across subjects (Bauer et al., 2015). Future studies might, therefore, consider measuring it as well, e.g., by applying questionnaires like the KVIQ (Malouin et al., 2007).

The findings of this study indicate that—in neurorehabilitation approaches on the basis of motor imagery and NMES—the stimulation parameters applied to maximize synaptic input to spinal circuitries should be carefully considered; in this context submaximal stimulation may improve the intended neurorehabilitation effects.

## Data Availability Statement

The data supporting the conclusions of this article will be made available by the authors, without undue reservation, to any qualified researcher.

## Ethics Statement

The studies involving human participants were reviewed and approved by the Ethics Committee of the Medical Faculty of the University of Tübingen. The patients/participants provided their written informed consent prior to participation in this study.

## Author Contributions

VR and RG: conceptualization, methodology, formal analysis, and writing – review and editing. AG: conceptualization, supervision, project administration, fund acquisition, and writing – original draft. All authors contributed to the article and approved the submitted version.

## Conflict of Interest

The authors declare that the research was conducted in the absence of any commercial or financial relationships that could be construed as a potential conflict of interest.
